# Blinding integrity of dorsomedial prefrontal intermittent theta burst stimulation in depression

**DOI:** 10.1016/j.ijchp.2023.100390

**Published:** 2023-05-12

**Authors:** Johan Bengtsson, Andreas Frick, Malin Gingnell

**Affiliations:** Department of Medical Sciences, Uppsala University, Akademiska Sjukhuset, Entrance 10, 3rd Floor, Uppsala 75185, Sweden

**Keywords:** RTMS, Blinding, Placebo, Sham

## Abstract

**Background:**

The antidepressant effect of repetitive transcranial magnetic stimulation (rTMS) is partly placebo, making blinding integrity important. Blinding of high-frequency rTMS and intermittent theta burst stimulation (iTBS) has been reported as successful at study end. However, blinding integrity at study start is rarely reported. The aim of this study was to investigate blinding integrity during a treatment course of iTBS over the dorsomedial prefrontal cortex (DMPFC) in depression.

**Methods:**

Forty-nine patients with depression from a double-blind-designed randomized controlled trial (NCT02905604) were included. Patients received either active or sham iTBS over the DMPFC with a placebo coil. The sham group received iTBS-synchronized transcutaneous electrical nerve stimulation.

**Results:**

After one session, 74% of participants were able to correctly guess their treatment allocation. This was above chance level (p = 0.001). The percentage dropped to 64% and 56% after the fifth and last sessions. Belonging to the active group influenced the choice to guess “active” (odds ratio: 11.7, 95% CI 2.5–53.7). A higher treatment intensity of the sham treatment increased the probability to guess “active”, but pain did not influence the choice.

**Conclusions:**

Blinding integrity in iTBS trials must be investigated at study start to avoid uncontrolled confounding. Better sham methods are needed.

## Introduction

Repetitive transcranial magnetic stimulation (rTMS) is an established treatment for major depressive disorder (Lefaucheur et al., 2020). However, there is recent critique against the use of rTMS. The arguments are that the evidence is based on small, underpowered studies, that the cumulative evidence is inconsistent, and that the antidepressant effect is modest and specific for a poorly defined group of patients, referred to as treatment-resistant, but lacking clinical characteristics such as melancholic features or psychotic symptoms (Malhi et al., 2021). Recent findings suggest that there is a high risk of bias in many existing systematic reviews (Brini et al., 2022). There is also a disproportionate number of significant findings, which might indicate publication bias (Amad et al., 2019). Furthermore, it has been suggested that a large proportion of the treatment effect is unspecific (Malhi & Bell, 2021) and the rTMS-associated placebo response has indeed been found to be large, with an effect size of 0.8 (Razza et al., 2018). Thus, sham protocols that ensure blinding integrity are of utmost importance. For dorsolateral high-frequency rTMS, there have been findings that participants cannot correctly guess their treatment allocation better than chance at study end, either in the active group or in the sham group (Berlim et al., 2013). However, the same meta-analysis observed an association between treatment response and the ability to correctly guess treatment allocation (Berlim et al., 2013). This observation has also been replicated in a later study (McGirr et al., 2021).

One of the most commonly used treatment protocols for major depressive disorder is intermittent theta burst stimulation (iTBS), which delivers burst of three pulses at theta frequency (Huang et al., 2005). A large non-inferiority trial without a placebo arm has paved the way for iTBS as a standard treatment protocol for depression (Blumberger et al., 2018). Regarding blinding of iTBS, it has been found that healthy volunteers can guess better than chance whether they have received sham stimulation over the motor cortex, but not if they have received active stimulation (Flanagan et al., 2019). Controlled trials of iTBS that assess blinding integrity often do so only at study end, which is not sufficient to be sure that early beliefs about treatment group have not influenced the results (Braga et al., 2021; Cole et al., 2022; Li et al., 2020; McGirr et al., 2021; Philip et al., 2019).

Hence, the aim of this study was to investigate the blinding integrity of iTBS over the dorsomedial prefrontal cortex (DMPFC) in depression, at both study start and end. Further aims were to identify factors that affected the participants’ guesses, and to assess whether the guesses affected treatment response.

## Material and methods

### Participants

**Fig. 1:**
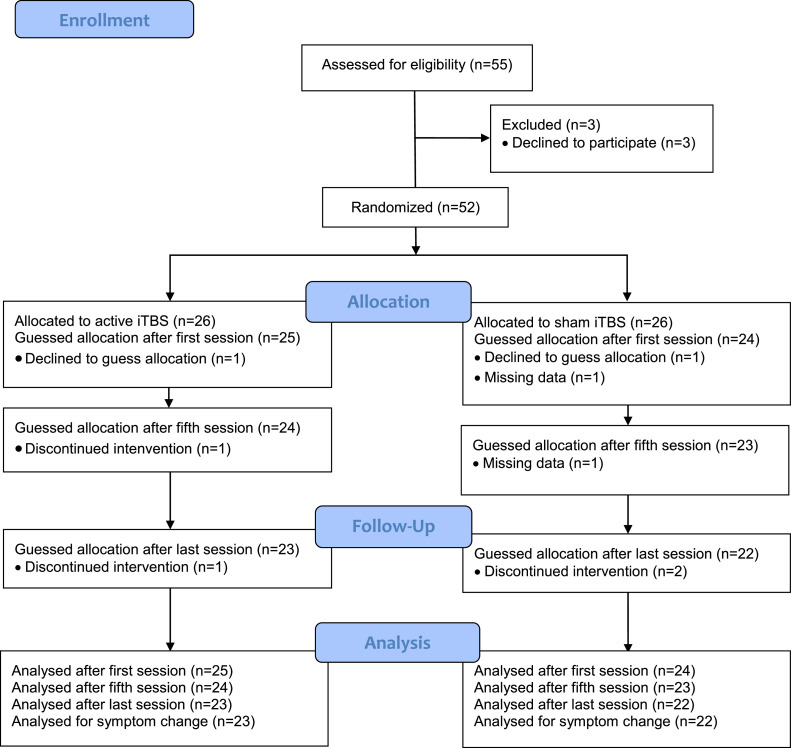
CONSORT flowchart of the subsample constituting the current study.

The study sample consisted of 49 patients who had participated in a pre-registered double-blind-designed randomized controlled trial (NCT02905604) or in an add-on brain-imaging study (Boden et al., 2021; Persson et al., 2020). The aim of the original trial was to evaluate the effectiveness of iTBS over the DMPFC for negative symptoms in patients with schizophrenia or depression. In the present study, we focused on depressive symptoms and thus only included the subset of patients with a depressive disorder verified through a Mini International Neuropsychiatric Interview (Sheehan et al., 1998), a score of 40 points or less on the Motivation and Pleasure Scale-Self-Report (Llerena et al., 2013), and an unchanged medication regimen during the past month. Exclusion criteria were contraindications to rTMS (metals implanted in the head, epilepsy, pacemakers, vagus nerve stimulators, medication pumps etc.), any condition with a substantial risk of non-compliance or loss to follow-up, such as active substance use disorder (except nicotine and caffeine), and pregnancy. See Fig. 1 for a CONSORT flowchart. Patients with schizophrenia (*n* = 13) were excluded as we wanted to obtain homogeneity of treatment indications. The trial was conducted at the unit for brain stimulation at Uppsala University Hospital. Patients were recruited from 2016 to 2020. All patients provided written informed consent and all procedures were in accordance with the Helsinki Declaration and approved by the national and institutional ethical committees.

### Design

Patients were screened at an initial visit and scheduled for a baseline visit if they were enrolled in the study. At the baseline visit, patients were interviewed with symptom assessment scales, filled in self-reported measures such as the Montgomery-Åsberg Depression Rating Scale – Self-report (MADRS-S) (Svanborg & Asberg, 2001), and underwent TMS resting motor threshold (RMT) determination. Treatment began on the following day and continued for 10–15 consecutive weekdays, depending on titration speed (see below). Follow-up symptom assessments were performed on the day after the last treatment session, and four weeks after the first treatment day. Treatment allocation was revealed at the second follow-up visit.

### rTMS procedures

The magnetic stimulator Magpro X100 with Magoption was used in this study. The RMT was determined by delivery of magnetic pulses with a Cool d-B80 coil over the medial primary motor cortex of the extensor hallucis longus. The lowest intensity needed to elicit a visually observable muscle contraction in 50% of the trials was set as the RMT, determined using an automated maximum likelihood strategy (Ah Sen et al., 2017).

For the treatment, a Cool d-B80 A/P coil was used. The coil is constructed with two identical sides, but the sham side is insulated, which prevents approximately 95% of the magnetic field from reaching the subject. For treatment allocation, the operator nurse fed the stimulator with a randomization code prepared by a contract research organization. The coil is equipped with a position sensor which enables the research software to direct the operator as to which side of the coil should be angled towards the subject.

**Fig. 2:**
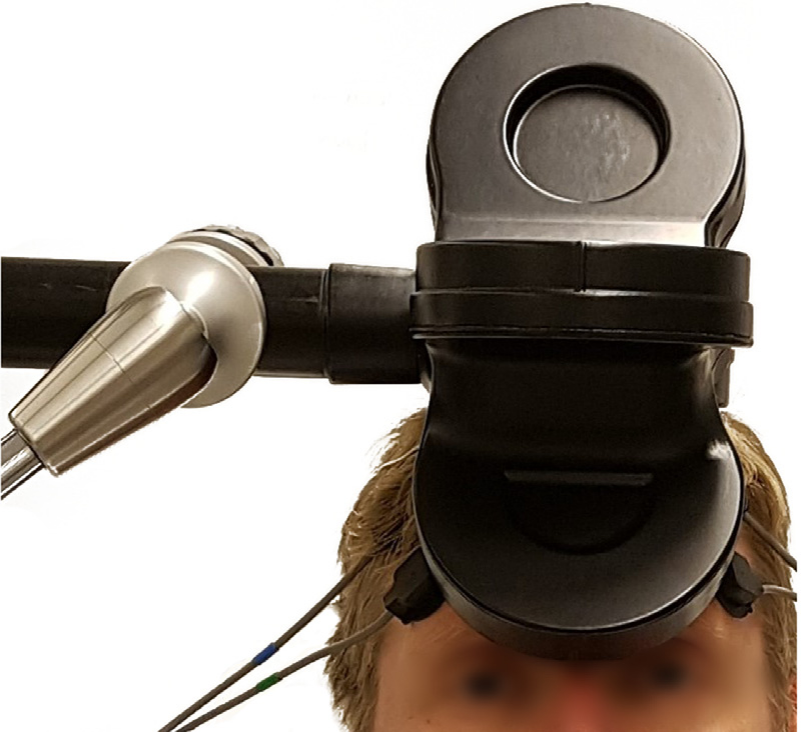
Coil positioning during treatment (courtesy of Janine Falk).

For the first 10 patients, the DMPFC treatment target was defined as 25% of the nasion-inion distance in the midline of the scalp. For those who also participated in the add-on brain imaging study (*n* = 39) (Persson et al., 2020), the DMPFC was located with a magnetic resonance imaging-based neuronavigational system (TMS Navigator, Localite, Bonn, Germany), with the following Montreal Neurological Institution coordinates: *x* = 0, *y* = 30, *z* = 30 (Mir-Moghtadaei et al., 2016). The coil was positioned tangentially to the scalp, with the handle pointing toward the right side of the patient. See Fig. 2 for a picture of coil positioning.

The active group received a modified version of the previously described iTBS protocols (Bakker et al., 2015; Chistyakov et al., 2010). The stimulation was applied at 90% of RMT, with 20 trains of right-left stimulation and 20 trains of left-right stimulation. Each train consisted of two seconds of stimulation, followed by eight seconds without stimulation. The stimulation comprised ten bursts at five Hertz and three biphasic pulses at 50 Hz within each burst, resulting in a total of 1200 pulses per session. To accelerate the treatment, a second identical treatment session was undertaken after a 15 min break (Duprat et al., 2016). The sham treatment comprised an identical stimulation protocol, but with the shielded side of the coil toward the patient. The auditory sensation was thus identical in both groups. For both groups, two transcutaneous electrical nerve stimulation (TENS) electrodes were also placed under the coil, medially on the forehead. During sham treatment, a current of a maximum of four milliamperes was delivered synchronously with the magnetic pulses in the iTBS protocol. The TENS device was directly connected to the magnetic stimulator and scaled to the output intensity.

For each participant, the treatment intensity was titrated up to reach target intensity at 90% of RMT. The patients themselves set the speed of the titration by verbally letting the operator know if he/she could increase the intensity. As the TENS device in the sham group was connected to the TMS device, the TENS could be titrated in the same manner. A session was regarded as complete when at least 50% of the trains reached target intensity. Up to five extra days of treatment were added to the whole treatment course if participants did not reach their target intensity. As a measure of treatment intensity, we computed which percentage of the individual's target magnetic stimulator output (MSO) that had been reached after the last train of the first treatment session, i.e., when the participant rated their pain and guessed treatment allocation (see below).

### Pain assessments

The patients rated their experienced pain immediately after the last train of the first, fifth, and final treatment sessions. Ratings were performed using a visual analogue scale (VAS) from 0 to 10. Zero was described as no pain and 10 as the worst imaginable pain.

### Guessing procedures

After having rated their pain on the first, fifth, and last treatment day, patients filled out a form where they guessed whether they had received active or sham stimulation. There was no “*Unsure*” alternative nor a confidence evaluation about their guesses. See the Limitations section for a discussion regarding this topic.

### Statistics

Data were assessed for normality by visual inspection of histograms and using the Shapiro-Wilks test. If variables were deemed to be normally distributed, means and standard deviations were reported, while if the data distributions were considered to be skewed, medians and inter-quartile ranges were reported. To investigate differences in demographic variables between the groups, we used the independent-samples *t*-test for continuous parametric variables, the Mann-Whitney U test for continuous non-parametric variables, and the chi-squared or Fisher's exact test for dichotomous and rank variables.

The percentages of participants who correctly guessed their treatment allocation were calculated. Deviations from chance levels were investigated with binomial tests and differences between the groups with chi-squared tests. The McNemar test was used to assess differences between the first and last session in both groups.

To assess which factors influenced the choice to guess that one had received the active treatment, a multiple logistic regression model was constructed with “guessing active” as the dependent variable and the following variables as predictors: MSO after the first session (i.e., treatment intensity), pain (VAS) experienced after the first session, and treatment allocation group (active or sham). Odds ratios (ORs) with 95% confidence intervals (CIs) were computed.

Symptom level reduction was investigated between baseline and the follow-up two weeks after the last treatment using a linear mixed model with symptoms as dependent variables and time and treatment group as predictors, using a maximum likelihood estimation with random intercept per subject. If the choice to guess active or sham, or the ability to correctly guess treatment allocation, influenced symptom reduction was also investigated with separate linear mixed models. Subjects were used as random effects and other variables were fixed. Statistical analyses were performed using JASP (JASP Team 2022, version 0.11.1) and SPSS (version 28).

## Results

### Participants

One participant in each group declined to guess treatment allocation. Two participants in each group discontinued the intervention and were excluded from the symptom reduction analyses, but included in the analyses of the guessing before discontinuation. See Fig. 1.

### Demographics and clinical characteristics

The two groups did not differ in age, sex, comorbidity, or educational or functional features. The active group had somewhat higher mean body mass index. Drug prescriptions were similar between the groups, as were symptom levels. The active group experienced more pain after the first session and did not reach their target intensity after the first session. See Table 1 for details.

**Table 1:** Demographic and clinical characteristics (n (%) unless otherwise stated).

	Active (*n* = 25)	Sham (*n* = 24)	p^a^
Age (years), median (IQR)	27 (13)	27 (11)	0.826
Sex, n (female/male)	14/11	13/11	0.897
BMI (kg/m^2^), mean (SD)	29 (7)	24 (6)	0.013
Bipolar depression	1 (4)	3 (13)	0.349
Comorbidity anxiety disorders	9 (36)	12 (50)	0.322
Comorbidity autism spectrum disorder	4 (16)	1 (4)	0.349
Comorbidity ADHD/ADD	3 (12)	7 (29)	0.171
Comorbidity personality disorder	2 (8)	3 (13)	0.667
Supported housing	2 (8)	5 (21)	0.247
Graduated from high school	22 (88)	19 (79)	0.403
Working or studying at least half-time	11 (44)	13 (54)	0.477
Sheehan Disability Scale, median (IQR)	19 (9)	19 (8)	0.888
Nicotine user^b^	7 (28)	8 (33)	0.686
AUDIT total score, median (IQR)	4 (3)	5 (5)	0.904
DUDIT total score, median (IQR)	0 (2)	0 (2)	0.950
Prescriptions			
SSRI	7 (28)	5 (21)	0.560
SNRI	7 (28)	8 (33)	0.686
TCA	5 (20)	2 (8)	0.417
Other antidepressant	12 (48)	8 (33)	0.296
Antidepressant combination	7 (28)	7 (29)	0.928
Mood stabilizer	6 (24)	6 (25)	0.935
Antipsychotic	5 (20)	5 (21)	0.942
Maudsley Staging Method, mean (SD)	10 (2)	11 (2)*	0.231
MADRS-S total score, mean (SD)	30 (8)	30 (7)	0.982
Motor threshold, mean (SD)	54 (10)	51 (10)	0.095
Pain after first session, VAS, mean (SD)	4.2 (1.8)	2.8 (2.2)	0.026
Percent of target intensity after first session, median (IQR)	90 (33)	100 (0)	0.002
Treatment days, mean (SD)	11.4 (2.6)	10.7 (1.7)	0.339

IQR = interquartile range, BMI = body mass index, SD = standard deviation, ADHD = attention deficit hyperactivity disorder, ADD = attention deficit disorder, AUDIT = Alcohol Use Disorders Identification Test, DUDIT = Drug Use Disorders Identification Test, SSRI = selective serotonin reuptake inhibitor, SNRI = serotonin and norepinephrine reuptake inhibitor, TCA = tricyclic antidepressant agent, MADRS-*S* = Montgomery Åsberg Depression Rating Scale self-rating, VAS = visual analogue scale.

a Independent-samples *t*-test/Mann-Whitney U test for continuous variables, chi-squared test for dichotomic variables, and Fisher's exact test if *n* < 5 in any cell with dichotomic variables.

b Tobacco or Swedish snuff.

⁎ One missing.

### Guessing

After the first treatment session, 74% of all participants were able to correctly guess their treatment allocation. This was significantly above chance level (*p* = 0.001). After the fifth and the last treatment session, we could no longer detect higher than chance level correct guessing, as the percentage dropped to 64% (*p* = 0.079) and 56% (*p* = 0.551), respectively. There were no significant differences between the groups regarding the proportions who guessed correctly, but significantly fewer participants in the active group correctly guessed their treatment allocation as the treatment period progressed (McNemar test, χ^2^=2.4, *p* = 0.039). No such difference was found in the sham group (McNemar test, χ^2^=0.4, *p* = 0.754). Thus, although they were in fact given real iTBS, the active group started to guess that they had received sham. See Table 2 for details.

**Table 2:** Percentage that correctly guessed treatment allocation.

	After first session	After five sessions	After last session
All	74% *(n = 49)*	64% *(n = 47)*	56% *(n = 45)*
Better than chance? *(binomial test)*	Yes *(p = 0.001)*	No *(p = 0.079)*	No *(p = 0.551)*
Active	72%	54%	44%*
Sham	75%	74%	68%*
Difference active/sham? *(chi-squared test)*	No χ*^2^=0.057* *(p = 0.812)*	No χ*^2^=1.984* *(p = 0.159)*	No χ*^2^=2.779* *(p = 0.095)*

⁎ Statistically different from first guess in the active group (McNemar test, χ^2^=2.4, *p* = 0.039) but not in the sham group (McNemar test, *χ*^2^=0.4, *p* = 0.754).

**Fig. 3:**
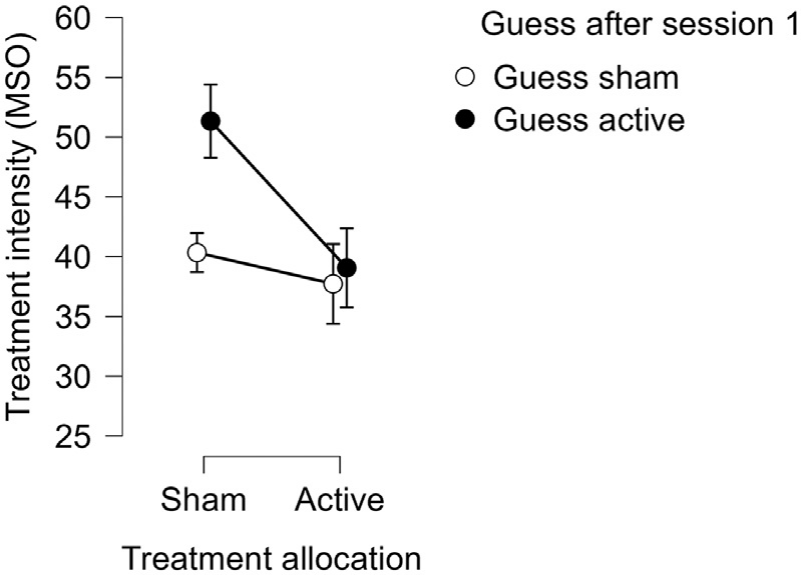
The interaction between treatment allocation and treatment intensity on guessing sham or active (MSO=Magnetic Stimulator Output).

We could not detect a robust relationship between the intensity of the treatment and the choice to guess active at the first (OR=1.1, 95% CI 1.0–1.1, *p* = 0.096) or last treatment session (OR=1.1, 95% CI 1.0–1.1, *p* = 0.336). The experienced pain did not affect the choice, either at the first (OR=1.0, 95% CI 0.7–1.4, *p* = 0.894) or the last treatment session (OR=1.1, 95% CI 0.8–1.5, *p* = 0.462). Treatment allocation group (i.e., active or sham) strongly affected the choice to guess that one had received active stimulation after the first treatment session (OR=11.7, 95% CI 2.5–53.7, *p* = 0.002), but not after the last (OR=1.4, 95% CI 0.4–5.0, *p* = 0.655). We found a moderating effect of the treatment intensity on the relationship between treatment allocation and guessing active (OR=0.8, 95% CI 0.6–1.0, *p* = 0.039). This could be explained by a relationship between treatment intensity and guessing active in the sham group, such that patients in the sham group who had higher treatment intensity more often guessed active (See Fig. 3). We could not find a moderating effect of pain on the relationship between treatment allocation and guessing active after the first treatment session (OR=0.6, 95% CI 0.3–1.2, *p* = 0.158).

### Symptom reduction

MADRS-S symptom levels decreased by 3.6 points (*p* = 0.064) between baseline and two weeks after the last treatment. There was no main effect of group (estimate < 0.1, *p* = 0.996) and no group☓time interaction effect (estimate=-0.7, *p* = 0.848) on symptom reduction. The choice to guess active or sham after the first treatment session did not influence symptom reduction (estimate=-0.7, *p* = 0.856), nor did the ability to correctly guess treatment allocation (estimate=-0.6, *p* = 0.889). The same was true for the guessing after the last treatment session (estimates -2.0, *p* = 0.602 and -0.1, *p* = 0.979, respectively). There were four participants receiving at least a 50% symptom reduction in the active group, and one in the sham group (a non-significant difference, χ^2^=2.0, *p* = 0.156). One participant in each group reached remission, defined as a total score on MADRS-S equal to 10 or less. Lastly, we did not detect an effect of treatment response on the choice to guess active or sham after the treatment (OR=0.94, 95% CI 0.85–1.0, *p* = 0.21).

## Discussion

In this follow-up investigation of rTMS treatment blinding integrity, we showed that 74% of participants receiving active or sham iTBS over the DMPFC could correctly guess their treatment allocation better than chance at study start, but not at the middle or end of treatment. This is an extension of previous studies reporting intact blinding at end of high-frequency rTMS treatment (Berlim et al., 2013). Our results can be compared with a study in healthy controls, where participants could guess better than chance that they had received sham iTBS stimulation, but not active (Flanagan et al., 2019). However, that study stimulated the motor hotspot at 60% of RMT with a straight figure-of-eight coil and used no TENS for the sham group, thus hampering further comparisons.

The experienced pain did not affect the choice to guess active or sham. However, in the sham group, a higher treatment intensity of the TENS increased the probability to guess that one had received active stimulation. It thus seems that the choice to guess that one has received active treatment depends on the differing sensations from the active iTBS and the TENS used in the sham group. This calls for better sham methods and our results indicate that one possible way forward could be to augment the intensity of the TENS if such a device is used. It could still be that it's not just the intensity of the TENS that matters, but other factors such as for example a qualitatively different sensation from iTBS. Increasing the intensity is however a quite easy and straight-forward solution to start with.

During the course of the treatment, the percentage who correctly guessed treatment allocation dropped in the active group, suggesting that something else also affected the participants’ guesses. Speculatively, the lack of treatment effect may influence these guesses, making participants in the active group doubt their initial (correct) belief and causing them to think that they are receiving sham. This shift in perceived group status could possibly also introduce a negative expectation effect, further hampering symptom reduction. The lack of a treatment effect, followed by a reduction in the capacity to make correct guesses in the active group, could also be viewed in the light of earlier findings of an association between clinical improvement and the ability to correctly guess treatment allocation (Berlim et al., 2013; McGirr et al., 2021). As rTMS is known to be highly susceptible to placebo effects (Razza et al., 2018), it might be the case that expectational factors are so important for driving the treatment responses in rTMS, that any doubts about treatment allocation are enough to abolish clinical improvement. In our sample, however, we did not detect an effect of symptom reduction on the guessing, but it should be noted that few participants actually had a treatment effect.

There is a striking underreporting of blinding integrity in rTMS RCTs (Berlim et al., 2013; Broadbent et al., 2011). Of the studies that report blinding, the majority investigate blinding integrity after the last treatment session. A meta-analysis from 2011 concluded that currently used sham methods were inadequate (Broadbent et al., 2011), and our results further support this notion.

The fact that participants in the active group perceived more pain after the first session, even if they did not reach their target intensity at the same rate, is one of the factors which could be improved in future sham treatments. Though the experienced pain did not seem to affect guessing, a proper sham condition should resemble the active stimulation as closely as possible in order not to compromise blinding integrity. Our finding that receiving active iTBS, but not TENS, affected guessing also calls for further development of the sham methods currently used.

A related question that would merit investigation is whether the RMT determination affects participants’ ability to correctly guess treatment allocation. In general, all participants in rTMS trials have experienced the sensation of real TMS pulses during the RMT determination, and although these single pulses over motor areas do not fully resemble those of rTMS protocols over prefrontal areas, it is possible that they affect participants’ ability to correctly differentiate between rTMS and TENS.

Our findings need to be replicated in larger groups and with a range of treatment protocols, locations, and coils to gain robustness. As rTMS is now an established treatment, ethical questions can hinder randomized studies. At the same time, our findings add to the critique of rTMS as an effective treatment. If blinding integrity is found to be lacking, there is a risk of uncontrolled bias of expectational effects in seemingly blinded-designed randomized controlled trials in the field (Braga et al., 2021), thus calling into question the results of these trials and, ultimately, the use of rTMS as an effective treatment. At a minimum, blinding integrity must be investigated at both study start and end in future RCTs. It is also questionable to implement iTBS as a standard treatment and to use non-inferiority studies (Blumberger et al., 2018) as evidence in clinical guidelines before this issue has been elucidated.

### Limitations

Some limitations need to be addressed. The sample consisted of 49 patients with depression receiving active or sham iTBS over the DMPFC with a Cool d-B80 A/P coil. The results are therefore not necessarily generalizable to other rTMS protocols, other treatment locations, or other coils. Further, symptom levels did not decrease significantly and data related to symptom reduction must therefore be interpreted with caution.

The treatment guessing did not include an *“Unsure”* alternative nor a confidence evaluation, which might have introduced bias. The placebo effect of a participant being strongly convinced may differ from a participant being unsure. However, there is also a risk that participants opt for the *“Unsure”* alternative falsely (i.e. even if they actually do have a sense of what arm they belong to), and this was the major reason for us to stick with just two choices. However, future studies could incorporate a confidence evaluation in order to address these issues and also adopt available blinding index assessments (Bang et al., 2010).

## Conclusions

Blinding integrity of iTBS was found to be broken already after the first treatment session. Existing trials of iTBS might therefore suffer from uncontrolled bias due to inefficient blinding. If this finding will hold true in future investigations it could have serious implications for the field. Future trials of rTMS must investigate blinding integrity also at study start. Further, better sham methods are needed to establish blinding integrity for iTBS. One possible way forward could be to increase the sham stimulation intensity or perhaps to refrain from the RMT determination in the sham group. These issues call for more and urgent research in order to avoid the expansion of a potentially non-blinded treatment.

## Funding information

The study was funded by research grants from the Swedish Research Council and the Märta and Nicke Nasvell Foundation. JB was supported by the Lennander Foundation. AF was supported by the Kjell and Märta Beijer Foundation.

## Declaration of Competing Interest

We have no known conflict of interest to disclose.
